# The value of mitigating epidemic peaks of COVID-19 for more effective public health responses

**DOI:** 10.1590/0037-8682-0135-2020

**Published:** 2020-03-23

**Authors:** Daniel Antunes Maciel Villela

**Affiliations:** 1 Fundação Oswaldo Cruz, Programa de Computação Científica, Rio de Janeiro, RJ, Brasil.

The emergence of SARS-Cov-2 virus in Wuhan, China, in December of 2019 led to a local epidemic that rapidly spread to multiple countries in the world, placing remarkable challenges in surveillance and control. In March 16^th^, 2020, WHO declared that the infection associated with SARS-Cov-2, named COVID-19, had spread to more than 100 countries, with more than 160,000 confirmed cases and more than 6,000 deaths globally^1^.

Great uncertainty remains regarding the epidemic peaks that might occur in various places where the virus has arrived due to factors such as environmental and social conditions and immunity levels of the population. These peaks are clearly related not only to the size of local populations but also to the disease reproduction number, which tells the mean number of secondary cases that are expected from an initial case in a susceptible population^2^. For public health stakeholders and their local surveillance teams to prepare mitigating strategies, an assessment of basic reproduction numbers can be quite helpful. Reproduction number estimates of COVID-19 have varied in different localities within ranges from 1.4 to 3.9 ^3^ ^-^ ^5^, with epidemic doubling time estimated at 6.4 days by Wu et al.^3^ and 7.4 days by Li et al.^4^ Clearly, peaks will also depend on eventual awareness of the local populations, prevention measures on surveillance and interventions taking place.

The epidemic curve that best fits the expected number of new cases in these localities shows a very large increasing rate in initial days and weeks, but followed by a gradual slow down until reaching an epidemic peak. Beyond this peak, the daily count of number of cases is expected to reduce, notwithstanding eventual adverse conditions causing ephemeral rebounds. Therefore, once an epidemic is already established, the first aim should be on reducing epidemic peaks. This will reduce the burden placed on health systems, which will permit proper treatment and care for the affected population. 

The number of new cases can be assessed by a product between the force of infection, a measure of its transmission intensity, clearly proportional to its prevalence, and the number of susceptible individuals^2^. In fact, infectious disease outbreaks eventually start to slow down because the number of susceptible individuals decreases. Therefore, an option would be to implement interventions to more effectively protect susceptible individuals. Ideally, such efforts would involve vaccination, which is not yet a real possibility at this moment for controlling the spread of SARS-Cov-2. 

Transmission intensity will also depend on the burden of viral infection and the time to clear such infection. Hence, another possibility is to apply interventions that reduce the time for virus clearance in infected people, decreasing transmission risk and, as a consequence, also decreasing the number of infected people. Such interventions for COVID-19 would require treatment as usually done for severe acute respiratory illnesses but problems still remain. First, there is potential transmission during virus incubation period, i.e., transmission before onset of symptoms when people typically will not enter treatment regimen, and the mean incubation period has been estimated at 5.2 days (95% CI: 4.1 - 7.0 days)^4^. Also, there is evidence of asymptomatic individuals capable of transmitting SARS-Cov-2, but the proportion of asymptomatic cases or how intense transmission could be from those cases remains an open question^6^. The fact is that transmission before symptoms or even in asymptomatic individuals makes control even harder. Second, targeted intervention would involve specific drug treatment regimens, which are not yet available due to the recent emergence of SARS-Cov-2. Furthermore, diagnostic tests, especially rapid ones, with appropriate levels of sensitivity and specificity will be quite helpful for implementing targeted interventions.

The force of infection also depends on the number of person-to-person interactions individuals have in their normal routines and also on how these interactions occur. At this moment, most recommendations for general population to avoid SARS-Cov-2 infection rely on altering social and individual routines, by implementing quarantine upon suspicion or detection of infection, by avoiding medium to large crowded events or gatherings, and also by applying regular hygiene practices or to establish norms for social interaction that avoid close contact. Also, discouraging travel reduces mobility and, as a consequence, the risk of importation and spreading of cases. Furthermore, upon detection of imported cases, personal isolation and contact tracing are also important to control new case bursts, but simulation results show that an effective control will require at least 70% of contacts to be traced, if the basic reproduction number equals 2.5 ^7^. Interestingly, Fraser et al. have shown that contact tracing also helps to determine the proportion of asymptomatic cases^8^.

Reducing the force of infection, however, can make the epidemic duration longer, even when reducing the peaks^9^ ^,^ ^10^. This happens for novel pathogens, such as SARS-Cov-2, since major portions of the population can be considered at risk or susceptible. These strict measures to avoid transmission reduce the peak of the epidemic, even if its duration takes longer, to daily or weekly levels which allows for health facilities to manage patient care and treatment. Therefore, reducing epidemic peaks avoids an overload on the health system to properly address the crisis. 

In terms of morbidity, attention is clearly needed to severe cases and the risk of death in people infected by SARS-Cov-2. The overall case fatality ratio of COVID-19, given by the ratio between the number of deaths and the number of confirmed cases, might appear small, but we observe that death cases concentrate on elderly individuals, when stratifying all cases leading to deaths by age groups. In Italy, among deceased people who were infected with SARS-Cov-2, more than 75% were aged 75 years or older ^11^. 

In countries with severe social disparities and health inequity, such as Brazil, major portions of the population receive suboptimal attention in the public health system. Given the urgency of the COVID-19 crisis, an important focus should also be placed on planning for more effective control to reduce the burden, especially severe cases and deaths, in the more vulnerable populations. 

## References

[1] World Health Organization (2020). Coronavirus disease 2019 (COVID-19) - Situation Report 56.

[2] Keeling MJ, Rohani P (2007). Modeling Infectious Diseases in Humans and Animals.

[3] Wu JT, Leung K, Leung GM (2020). Nowcasting and forecasting the potential domestic and international spread of the 2019-nCoV outbreak originating in Wuhan, China: a modelling study. The Lancet.

[4] Li Q, Guan X, Wu P, Wang X, Zhou L, Tong Y (2020). Early Transmission Dynamics in Wuhan, China, of Novel Coronavirus-Infected Pneumonia. N Engl J Med.

[5] Zhang S, Diao M, Yu W, Pei L, Lin Z, Chen D (2020). Estimation of the reproductive number of novel coronavirus (COVID-19) and the probable outbreak size on the Diamond Princess cruise ship: A data-driven analysis. Int J Infect Dis.

[6] Rothe C, Schunk M, Sothmann P, Bretzel G, Froeschl G, Wallrauch C (2020). Transmission of 2019-nCoV Infection from an Asymptomatic Contact in Germany. N Engl J Med.

[7] Hellewell J, Abbott S, Gimma A, Bosse NI, Jarvis CI, Russell TW (2020). Feasibility of controlling COVID-19 outbreaks by isolation of cases and contacts. Lancet Glob Health.

[8] Fraser C, Riley S, Anderson RM, Ferguson NM (2004). Factors that make an infectious disease outbreak controllable. Proc Natl Acad Sci.

[9] Adiga A, Chu S, Eubank S, Kuhlman CJ, Lewis B, Marathe A (2018). Disparities in spread and control of influenza in slums of Delhi: findings from an agent-based modelling study. BMJ Open.

[10] Anderson RM, Heesterbeek H, Klinkenberg D, Hollingsworth TD (2020). How will country-based mitigation measures influence the course of the COVID-19 epidemic?. The Lancet.

[11] Remuzzi A, Remuzzi G (2020). COVID-19 and Italy: what next?. The Lancet.

